# Deletion of Interleukin-4 Receptor Alpha-Responsive Keratinocytes in BALB/c Mice Does Not Alter Susceptibility to Cutaneous Leishmaniasis

**DOI:** 10.1128/IAI.00710-18

**Published:** 2018-11-20

**Authors:** Melissa Govender, Ramona Hurdayal, Berenice Martinez-Salazar, Kaya Gqada, Shandre Pillay, Lorna Gcanga, Katiuska Passelli, Natalie E. Nieuwenhuizen, Fabienne Tacchini-Cottier, Reto Guler, Frank Brombacher

**Affiliations:** a International Center for Genetic Engineering and Biotechnology (ICGEB), Cape Town Component, Cape Town, South Africa; b Institute of Infectious Diseases and Molecular Medicine (IDM), Department of Pathology, Division of Immunology, and South African Medical Research Council (SAMRC) on Immunology of Infectious Diseases, Faculty of Health Sciences, University of Cape Town, Cape Town, South Africa; c Wellcome Center for Infectious Diseases Research in Africa, Institute of Infectious Diseases and Molecular Medicine (IDM), Faculty of Health Sciences, University of Cape Town, Cape Town, South Africa; d Department of Molecular and Cell Biology, University of Cape Town, Cape Town, South Africa; e Department of Biochemistry, and WHO Immunology Research and Training Collaborative Center, University of Lausanne, Epalinges, Switzerland; f Department of Immunology, Max Planck Institute for Infection Biology, Berlin, Germany; g Department of Clinical and Experimental Medicine, Division of Molecular Virology, Linköping University, Linköping, Sweden; University of Pennsylvania

**Keywords:** IL-4 receptor alpha signaling, keratinocytes, *Leishmania major*, skin

## Abstract

The skin microenvironment at the site of infection plays a role in the early events that determine protective T helper 1/type 1 immune responses during cutaneous leishmaniasis (CL) infection. During CL in nonhealing BALB/c mice, early interleukin-4 (IL-4) can instruct dendritic cells for protective Th1 immunity. Additionally, keratinocytes, which are the principal cell type in the skin epidermis, have been shown to secrete IL-4 early after Leishmania major infection. Here, we investigated whether IL-4/IL-13 signaling via the common IL-4 receptor alpha chain (IL-4Rα) on keratinocytes contributes to susceptibility during experimental CL. To address this, keratinocyte-specific IL-4Rα-deficient (KRT14^cre^ IL-4Rα^−/lox^) mice on a BALB/c genetic background were generated by gene targeting and site-specific recombination (Cre/*loxP*) under the control of the keratinocyte-specific *krt14* locus. Following high-dose infection with L. major IL-81 and LV39 promastigotes subcutaneously in the footpad, footpad swelling, parasite burden, IFN-γ/IL-4/IL-13 cytokine production, and type 1 and type 2 antibody responses were similar between KRT14^cre^ IL-4Rα^−/lox^ and littermate control IL-4Rα^−/lox^ BALB/c mice. An intradermal infection with low-dose L. major IL-81 and LV39 promastigotes in the ear showed results in infected KRT14^cre^ IL-4Rα^−/lox^ BALB/c mice similar to those of littermate control IL-4Rα^−/lox^ BALB/c mice, with the exception of a significant decrease observed in parasite burden only at the site of LV39 infection in the ear. Collectively, our results show that autocrine and paracrine signaling of IL-4/IL-13 through the IL-4Rα chain on keratinocytes does not influence the establishment of a nonhealing Th2 immune response in BALB/c mice during L. major infection.

## Abstract (precis)

The skin microenvironment at the site of infection plays a role in the early events that determine protective T helper 1/type 1 immune responses during cutaneous leishmaniasis (CL) infection. During CL in nonhealing BALB/c mice, early interleukin-4 (IL-4) can instruct dendritic cells for protective Th1 immunity.

## INTRODUCTION

Murine studies in leishmaniasis provide a well-established model to investigate the T helper (Th) Th1/Th2 paradigm observed during Leishmania major infection. While a polarized Th1 immune response is associated with host protective immunity to L. major infection, a polarized Th2 immune response is affiliated with susceptibility to the disease (1–3). Th1 immunity during L. major infection is characterized by classical activation of macrophages via the cytokines interferon gamma (IFN-γ) and interleukin-12 (IL-12), while Th2 immunity is characterized by alternative activation of macrophages via the production of various cytokines, including IL-13, IL-5, and, primarily, IL-4, which signals via the IL-4 receptor alpha chain (IL-4Rα). Previous studies have demonstrated that a resistant phenotype was observed in C57BL/6 mice (healer strain) infected with L. major, while BALB/c mice (nonhealer strain) were susceptible to cutaneous leishmaniasis (CL) (2, 4–7). While the general understanding is that IL-4 induces a Th2 response detrimental to CL, there have been studies demonstrating that the early production of IL-4 at the site of infection in BALB/c mice drives a beneficial Th1 response under the instruction of dendritic cells (DCs) (8, 9). This phenomenon is further supported by the fact that both BALB/c and C57BL/6 mouse strains secrete IL-4 early after L. major infection, which is sustained in susceptible but transient in resistant mice (10). The skin, which serves as an immune organ (11), is the primary site of infection during cutaneous leishmaniasis (1). During a blood feed, the female phlebotomine sandfly deposits L. major promastigotes into the skin. The promastigote parasites must pass through this skin barrier and its components to establish an infection. The epidermal layer of the skin is composed primarily of keratinocytes, which produce factors such as cytokines, among others (12). Thus, keratinocytes could provide early signals at the site of L. major infection to initiate distinct immune effector responses. Indeed, infection with L. major IL-81 promastigotes has been shown to induce keratinocytes to rapidly secrete IL-12, IL-1β, and IL-4 in C57BL/6 mice. This suggests that keratinocytes provide the source of early IL-4 that may instruct DCs to drive the host beneficial Th1/type 1 response (13). As keratinocytes express surface IL-4 receptor, these cells are capable of both autocrine and paracrine stimulation (14, 15). We recently demonstrated that C57BL/6 mice deficient for IL-4Rα-responsive keratinocytes were able to develop a protective Th1/type 1 effector response to L. major LV39 infection (16). However, considering that the impact of IL-4-mediated DC instruction was most pronounced in the susceptible BALB/c background in response to more virulent and less virulent strains of parasites, the role of early IL-4 signaling on keratinocytes needs to be investigated on a nonhealer BALB/c genetic background during cutaneous leishmaniasis to fully elucidate effector immune responses in response to infection with more virulent and less virulent L. major strains. Here, we extended our recent study by generating keratinocyte-specific IL-4Rα-deficient mice on a BALB/c genetic background (KRT14^cre^ IL-4Rα^−/lox^ mice) to analyze disease progression and host immune responses following infection with the L. major strain IL-81 (a highly virulent strain) as well as LV39 (less virulent strain). We successfully showed that the IL-4Rα signal on keratinocytes from KRT14^cre^ IL-4Rα^−/lox^ BALB/c mice was absent, in contrast to the results for wild-type BALB/c mice. We found that during experimental cutaneous leishmaniasis, KRT14^cre^ IL-4Rα^−/lox^ BALB/c mice were more susceptible to infection, similar to littermate control IL-4Rα^−/lox^ BALB/c mice, following subcutaneous (s.c.) infection in the footpad or intradermal (i.d.) infection in the ear. Furthermore, footpad swelling, parasite loads, IFN-γ/IL-4/IL-13 production, and type 1 and type 2 antibodies were similar between both groups. Despite a significant decrease in parasite burden seen at the site of infection after i.d. inoculation of L. major LV39, KRT14^cre^ IL-4Rα^−/lox^ mice on the BALB/c genetic background still developed a nonhealing response. Taking our results together, we revealed that deletion of IL-4Rα signaling on keratinocytes does not influence susceptibility of genetically susceptible BALB/c mice to CL.

## RESULTS

### Genotypic and functional characterization of KRT14^cre^ IL-4Rα^−/lox^ BALB/c mice.

**FIG 1:**
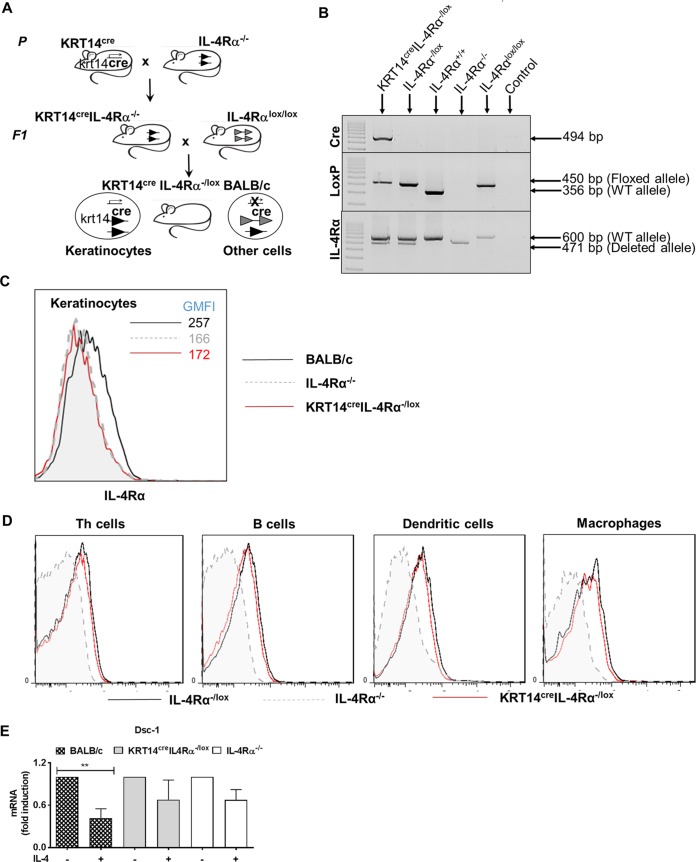
Characterization of KRT14^cre^ IL-4Rα^−/lox^ BALB/c mice. (A) Mouse breeding strategy. Transgenic BALB/c mice expressing cre-recombinase under the control of the KRT14 promoter were intercrossed with IL-4Rα^−/−^ BALB/c mice and IL-4Rα^lox/lox^ BALB/c mice to generate KRT14^cre^ IL-4Rα^−/lox^ mice. (B) Genotyping by PCR analysis of tail DNA from KRT14^cre^ IL-4Rα^−/lox^, IL-4Rα^−/lox^, IL-4Rα^+/+^, IL-4Rα^−/−^, and IL-4Rα^lox/lox^ mice and a negative water control is shown. The yielded PCR products are indicated in base pairs. (C) Flow cytometry was performed to show IL-4Rα expression on ear keratinocytes isolated from naive mice. Keratinocytes were gated as CD45^−^ CD49^+^ K14^+^. (D) Flow cytometry was performed to show IL-4Rα expression on nonkeratinocyte lymph node cells following L. major infection, staining for Th cells (CD3^+^ CD4^+^), B cells (CD19^+^ B220^+^), dendritic cells (CD11c^+^ MHCII^+^), and macrophages (CD11b^+^ MHCII^+^). (E) *Dsc-1* mRNA expression in keratinocytes. Primary keratinocytes were isolated from tails of adult BALB/c, KRT14^cre^ IL-4Rα^−/lox^, and IL-4Rα^−/−^ mice. Keratinocytes were left unstimulated (−) or stimulated (+) for 24 h with 20 ng/ml of recombinant IL-4. Cells were isolated and Dsc-1 mRNA expression was assessed via qRT-PCR. Values were normalized to *hprt* levels (*n* = 3 in each group; representative of two individual experiments shown). Statistical analysis for the mRNA expression of *Dsc-1* in keratinocytes was performed using a one-way analysis of variance (ANOVA), with Sidaks’s multiple-comparison test. **, *P* < 0.01.

Genetically modified BALB/c mice expressing Cre-recombinase under the control of the keratinocyte cell-specific locus *krt14* (Jackson Laboratory) were intercrossed with IL-4Rα^−/−^ BALB/c mice (17) and IL-4Rα^lox/lox^ BALB/c mice (18) to generate KRT14^cre^ IL-4Rα^−/lox^ mice (Fig. 1A). This breeding strategy avoids possible non-Mendelian Cre activities during early embryogenesis by reducing the substrate (*lox*), resulting in increased Cre efficiency (19, 20). KRT14^cre^ IL-4Rα^−/lox^ BALB/c mice were identified by PCR genotyping (Fig. 1B), as indicated by the presence of the 494-bp Cre band, 450-bp LoxP band, and both the deleted and wild-type IL-4Rα allele. Analysis of IL-4Rα cell surface expression on isolated ear keratinocytes (CD45^−^ CD49^+^ K14^+^) by flow cytometry demonstrated efficient IL-4Rα depletion on KRT14^cre^ IL-4Rα^−/lox^ keratinocytes, which was apparent by the shift of IL-4Rα expression and geometric mean fluorescence intensity (GMFI) compared to that of wild-type BALB/c keratinocytes (Fig. 1C). As expected, KRT14^cre^ IL-4Rα^−/lox^ mice were confirmed to have a regular cell surface expression of IL-4Rα on CD4^+^ T cells, CD19^+^ B cells, MHCII^hi^ CD11c^hi^ dendritic cells, and MHCII^hi^ CD11c^−^ CD11b^hi^ macrophages in the cervical lymph node after L. major infection (Fig. 1D). The expression of Desmocollin-1 (*Dsc-1*), a marker gene for keratinocytes, can correlate with normal epidermal differentiation and development of keratinocytes (21). Additionally, the presence of IL-4 has been shown to downregulate the expression of *Dsc-1* (22, 23). We therefore examined whether keratinocyte function would be altered due to loss of IL-4Rα on keratinocytes by measuring *Dsc-1* expression and treating with recombinant IL-4. IL-4 stimulation reduced mRNA expression of *Dsc-1* in wild-type BALB/c but not keratinocytes isolated from KRT14^cre^ IL-4Rα^−/lox^ or IL-4Rα^−/−^ mice, confirming functionally unresponsive IL-4Rα signaling in KRT14^cre^ IL-4Rα^−/lox^ keratinocytes (Fig. 1E). These data provide evidence of efficient deletion of IL-4Rα on keratinocytes from KRT14^cre^ IL-4Rα^−/lox^ BALB/c mice while showing intact IL-4Rα surface expression on other lymph node cells and demonstrating no effect on keratinocyte functionality.

### KRT14^cre^ IL-4Rα^−/lox^ BALB/c mice remain susceptible to L. major infection, similar to littermate control IL-4Rα^−/lox^ BALB/c mice, during experimental cutaneous leishmaniasis in the footpad.

**FIG 2:**
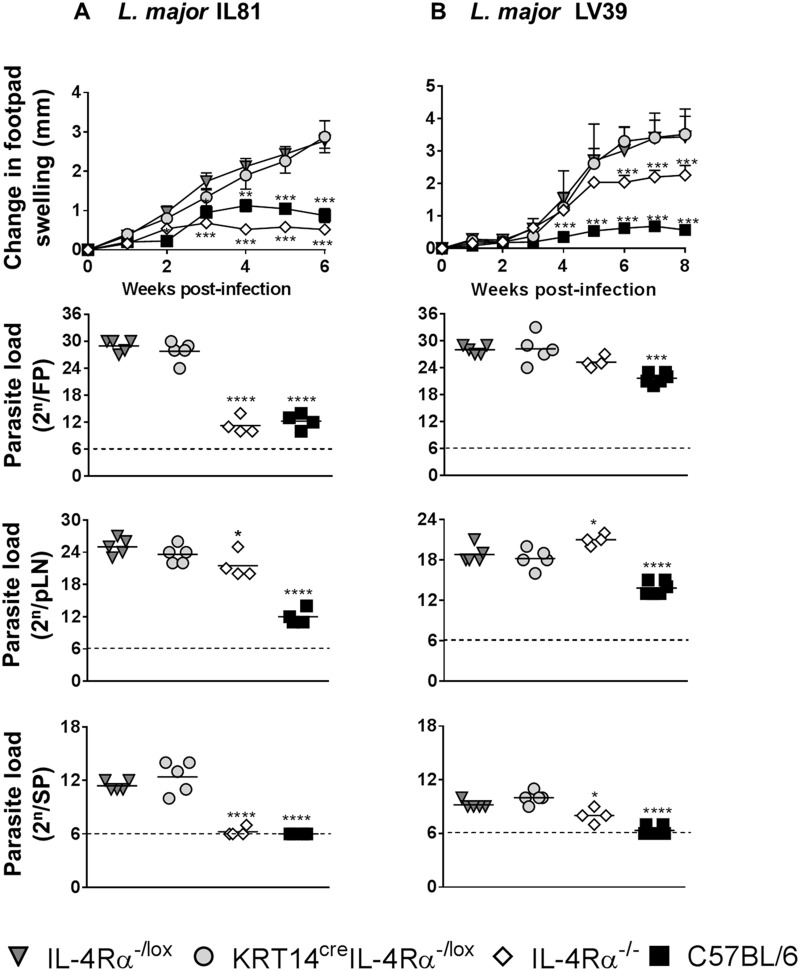
KRT14^cre^ IL-4Rα^−/lox^ BALB/c mice are as susceptible to L. major as littermate control mice following footpad infection. Mice were infected subcutaneously in the left hind footpad (FP) with stationary-phase L. major promastigotes at either 2 × 10^5^ IL-81 (A) or 2 × 10^6^ LV39 (B) (*n* = 5 to 7 mice per group). The change in footpad swelling (in mm) was measured at weekly intervals. Parasite burden was determined at week 6 for panel A and week 8 for panel B by limiting dilution of homogenized footpads, single-cell suspensions of the draining popliteal lymph nodes (pLN), and homogenized spleens (SP). A representative of two individual experiments is shown with mean values ± SEM. Statistical analysis was performed with comparisons to the control IL-4Rα^−/lox^ littermate mouse group as significant (*, *P* < 0.05; **, *P* < 0.01; ***, *P* < 0.001; ****, *P* < 0.0001), using a two-way ANOVA with Bonferroni posttests for change in swelling data and one-way ANOVA with Dunnett’s multiple-comparison test for parasite load data.

To determine whether IL-4Rα signaling on keratinocytes at the site of infection contributes to nonhealing disease during CL, KRT14^cre^ IL-4Rα^−/lox^ BALB/c mice and appropriate controls (IL-4Rα^−/lox^ BALB/c, IL-4Rα^−/−^ BALB/c, and C57BL/6 mice) were infected s.c. in the left hind footpad with stationary-phase promastigotes, either with 2 × 10^5^ of the highly virulent L. major IL-81 strain (Fig. 2A) or with a dose of 2 × 10^6^ of the less virulent L. major LV39 strain (Fig. 2B). Importantly, during both L. major IL-81 and L. major LV39 infections, KRT14^cre^ IL-4Rα^−/lox^ BALB/c mice developed progressive footpad swelling, similar to littermate control IL-4Rα^−/lox^ BALB/c mice, and exhibited similarly high parasite loads in the infected footpads and draining popliteal lymph nodes and dissemination to the spleen (Fig. 2). Global IL-4Rα^−/−^ BALB/c mice, which are generally resistant to L. major infection, had significantly less footpad swelling than littermate control IL-4Rα^−/lox^ BALB/c mice during infection with either strain (Fig. 2A). After infection with L. major IL-81, parasite loads of global IL-4Rα^−/−^ BALB/c mice in the footpad, popliteal lymph node, and spleen appeared to be significantly lower than the loads in littermate control IL-4Rα^−/lox^ BALB/c mice (Fig. 2A). Upon infection with L. major LV39, the parasite loads in the popliteal lymph nodes of global IL-4Rα^−/−^ BALB/c mice appeared to be significantly higher than those of the littermate control IL-4Rα^−/lox^ BALB/c mice, which has been observed previously (24), while parasite loads in the spleen were significantly lower, as anticipated (Fig. 2B). As expected, genetically resistant C57BL/6 mice controlled the development of lesions during acute infection with L. major IL-81 and LV39 (Fig. 2A and B), correlating with low parasite loads in footpads, draining popliteal lymph nodes, and spleens. Together, these results suggest that deletion of the IL-4Rα chain on keratinocytes in BALB/c mice does not affect L. major disease progression when parasites are subcutaneously injected into the footpad.

### Absence of IL-4Rα on keratinocytes in BALB/c mice has no functional effect on cellular and humoral immune responses during L. major IL-81 infection in the footpad.

**FIG 3:**
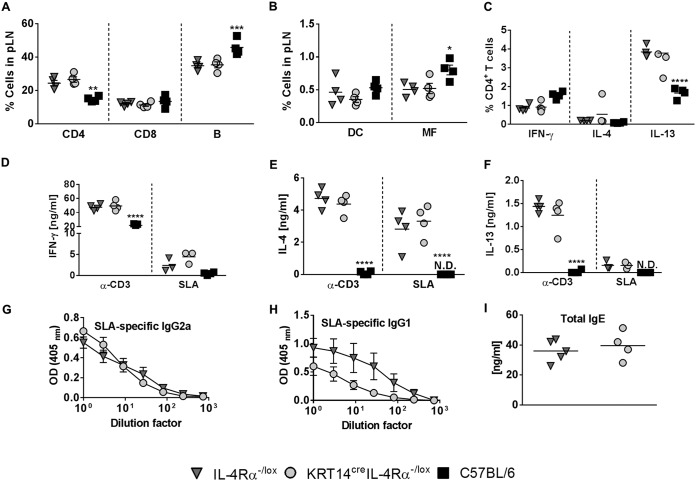
KRT14^cre^ IL-4Rα^−/lox^ BALB/c mice develop an immune response similar to that of littermate control mice following L. major IL-81 infection in the footpad. At 6 weeks after L. major IL-81 infection, mice were sacrificed and popliteal draining lymph nodes (pLN) retrieved. Single-cell suspensions obtained from the lymph nodes were surface stained for the following populations: CD4 (CD3^+^ CD4^+^) T cells, CD8 (CD3^+^ CD8^+^) T cells, and B cells (CD19^+^ B220^+^) (A) and DC (dendritic cells) (CD11c^hi^ MHCII^hi^) and MF (macrophages) (CD11b^hi^ CD11c^−^ MHCII^hi^) (B). (C) Lymph node cells were stimulated with PMA-ionomycin-monensin, followed by staining for intracellular cytokine production. (D to F) Cells were restimulated with anti-CD3 and soluble leishmanial antigen (SLA) for 72 h, after which cytokine production was measured with ELISA for (D) IFN-γ, (E) IL-4 and (F) IL-13. (G) L. major-specific type 1 (IgG2a) and (H and I) type 2 antibodies (IgG1 and total IgE) were measured in sera from 6-week infected mice by ELISA. Data are representative of two experiments with mean values ± SEM (*n* = 3 to 5 mice per group). Statistical analysis was performed with comparisons to the control IL-4Rα^−/lox^ littermate mouse group as significant (*, *P* < 0.05; **, *P* < 0.01; ***, *P* < 0.001; ****, *P* < 0.0001), using 2-way ANOVA with Dunnett’s multiple-comparison test (A to F), two-way ANOVA with Bonferroni posttests (G and H) and Mann-Whitney (nonparametric, unpaired *t*) test (I).

BALB/c mice are genetically susceptible to L. major infection and develop a detrimental type 2 immune response (2, 25). We therefore evaluated the cellular and humoral immune response in KRT14^cre^ IL-4Rα^−/lox^ BALB/c mice during L. major IL-81 infection to determine whether these mice elicited a polarized Th2 immune response. At 6 weeks postinfection, the frequencies of immune cell populations infiltrating the popliteal draining lymph nodes were similar between KRT14^cre^ IL-4Rα^−/lox^ BALB/c and the littermate control IL-4Rα^−/lox^ BALB/c mice, as determined by flow cytometry and cell surface staining (Fig. 3A and B). C57BL/6 mice at week 6 presented lower percentages of the CD3^+^ CD4^+^ Th cells, possibly due to disease control at this stage, and higher CD19^+^ B lymphocytes and macrophages than susceptible littermate control IL-4Rα^−/lox^ BALB/c mice (Fig. 3A and B). Collectively, these results suggest that the immune cell repertoire in the popliteal lymph node developed independently of IL-4Rα-responsive keratinocytes during L. major infection in KRT14^cre^ IL-4Rα^−/lox^ BALB/c mice.

To investigate the impact of IL-4Rα deficiency on keratinocytes in cytokine production by CD4^+^ Th cells during L. major IL-81 infection, single lymph node cell suspensions were restimulated with phorbol myristate acetate (PMA)-ionomycin-monensin and stained for intracellular cytokine production by flow cytometry (Fig. 3C). KRT14^cre^ IL-4Rα^−/lox^ BALB/c mice showed frequencies of IFN-γ/IL-4/IL-13 cytokines produced by CD4^+^ Th cells similar to those of littermate control IL-4Rα^−/lox^ BALB/c mice (Fig. 3C). As previously reported (9), resistant C57BL/6 mice had higher production of IFN-γ (although not significant) and significantly lower production of IL-13 by CD4^+^ Th cells than the littermate control IL-4Rα^−/lox^ BALB/c mice (Fig. 3C) following IL-81 infection in the footpad. Total popliteal lymph node cells were also restimulated with soluble leishmanial antigen (SLA) or mitogenic anti-CD3 to detect overall cytokine production by the immune cell repertoire (Fig. 3D to F). Similar levels of production of IFN-γ (Fig. 3D), IL-4 (Fig. 3E), and IL-13 (Fig. 3F) were observed in KRT14^cre^ IL-4Rα^−/lox^ BALB/c and littermate control IL-4Rα^−/lox^ BALB/c mice. In contrast, C57BL/6 mice showed significantly lower production of IFN-γ and drastically lower quantities of Th2 cytokines IL-4/IL-13 than littermate control IL-4Rα^−/lox^ BALB/c mice when lymph node cells were stimulated with anti-CD3 (Fig. 3D to F), as previously observed (26). Together, these results suggest that IL-4Rα-responsive keratinocytes do not play a decisive role in driving a polarized Th2 response during L. major infection in BALB/c mice in the footpad.

The quantification of cytokine production after *ex vivo* stimulation may not provide a true indication of the type 1 and type 2 or Th1 and Th2 responses *in vivo* (25). As it is known that IL-4 promotes isotype switching to IgG1 and IgE and that IgG2a levels correlate with the activity of IFN-γ *in vivo* (27), we measured antigen-specific type 1 (IgG2a) and type 2 (IgG1 and total IgE) antibody titers in the mouse sera by enzyme-linked immunosorbent assay (ELISA) 6 weeks postinfection with L. major IL-81. KRT14^cre^ IL-4Rα^−/lox^ BALB/c and littermate control IL-4Rα^−/lox^ BALB/c mice had comparable levels of IgG2a, IgG1, and total IgE during L. major IL-81 infection (Fig. 3G to I). Collectively, the data suggest that systemic antibody responses in KRT14^cre^ IL-4Rα^−/lox^ BALB/c mice were unaffected by the deletion of the IL-4Rα signaling receptor on keratinocytes during subcutaneous infection with L. major IL-81 in the footpad.

### Intradermal inoculation of L. major parasites in the ear does not radically alter the outcome of disease in KRT14^cre^ IL-4Rα^−/lox^ BALB/c mice.

**FIG 4:**
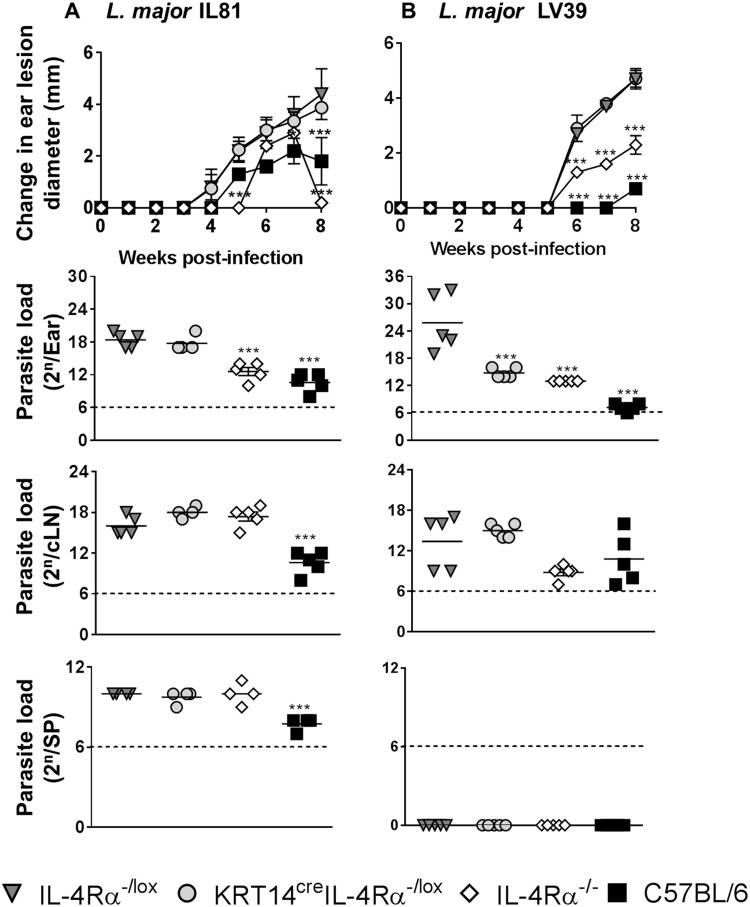
Cutaneous leishmaniasis is unaffected by the absence of IL-4Rα signaling on keratinocytes on a BALB/c genetic background following low-dose L. major infection in the ear dermis. Mice were infected intradermally in the left ear with 1 × 10^4^ promastigotes of L. major IL-81 (A) or L. major LV39 (B) (*n* = 5 to 7 mice per group). The change in ear lesion diameter (mm) was measured at weekly intervals. Parasite burden was determined at week 8, by limiting dilution of homogenized ear cells, single-cell suspensions of the draining cervical lymph nodes (cLN), and homogenized spleens (SP). A representative of two individual experiments is shown with mean values ± SEM. Statistical analysis was performed with comparisons to the control IL-4Rα^−/lox^ littermate mouse group as significant (*, *P* < 0.05; ***, *P* < 0.001), using a two-way ANOVA with Bonferroni posttests for change in ear lesion data and one-way ANOVA with Dunnett’s multiple-comparison test for parasite load data.

While s.c. injection of L. major in the footpad is widely used, recent studies have suggested that i.d. injection of a lower dose of parasites into the ear provides a more physiological mode of infection, as it mimics some of the events that occur during parasite inoculation by the sandfly (28, 29). Hence, we investigated whether a change in the site of infection and parasite dose would alter the phenotype and immune response of the KRT14^cre^ IL-4Rα^−/lox^ BALB/c mice. Mice were i.d. infected into the left ear with a low dose of 1 × 10^4^ stationary-phase L. major IL-81 or L. major LV39 promastigotes. Ear lesion diameter was measured to monitor disease progression during the infection, and immune responses were evaluated at 8 weeks postinfection. Ear lesion diameter and the parasite load in the ear, cervical lymph node, and spleen of KRT14^cre^ IL-4Rα^−/lox^ BALB/c mice during L. major IL-81 infection were high, similar to those of littermate control IL-4Rα^−/lox^ BALB/c mice (Fig. 4A). Global IL-4Rα^−/−^ BALB/c mice had significantly smaller lesion diameters and lower parasite loads in the ear than littermate control IL-4Rα^−/lox^ BALB/c mice after infection with L. major IL-81 (Fig. 4A). As previously reported (30), C57BL/6 mice had significantly smaller lesion diameters and associated reduced parasite load in the ear, cervical lymph node, and spleen compared to littermate control IL-4Rα^−/lox^ BALB/c mice after L. major IL-81 infection (Fig. 4A). Infection with L. major LV39 resulted in similar ear lesion diameters and cervical lymph node parasite loads between KRT14^cre^ IL-4Rα^−/lox^ BALB/c and littermate control IL-4Rα^−/lox^ BALB/c mice, with the surprising exception of significantly reduced parasite loads in the ear (Fig. 4B). Both global IL-4Rα^−/−^ BALB/c and C57BL/6 mouse groups had significantly smaller lesion diameters in the ears and significantly lower parasite loads in the ear than littermate control IL-4Rα^−/lox^ BALB/c mice after L. major LV39 infection (Fig. 4B). In contrast to L. major IL-81 infection, L. major LV39 infection did not result in dissemination to the spleen, as demonstrated by the absence of parasites in the spleen in all mouse groups. This could be explained by the higher virulence of the former parasite (9) and the i.d. route of infection. Collectively, the data indicate that despite a change in the site/route of infection and the dose of parasites, KRT14^cre^ IL-4Rα^−/lox^ BALB/c mice remain susceptible to L. major and remain unaffected by the abrogated IL-4Rα signaling on keratinocytes.

### Early IL-4 production in the ear appears unaffected by loss of signaling of IL-4Rα on keratinocytes.

**FIG 5:**
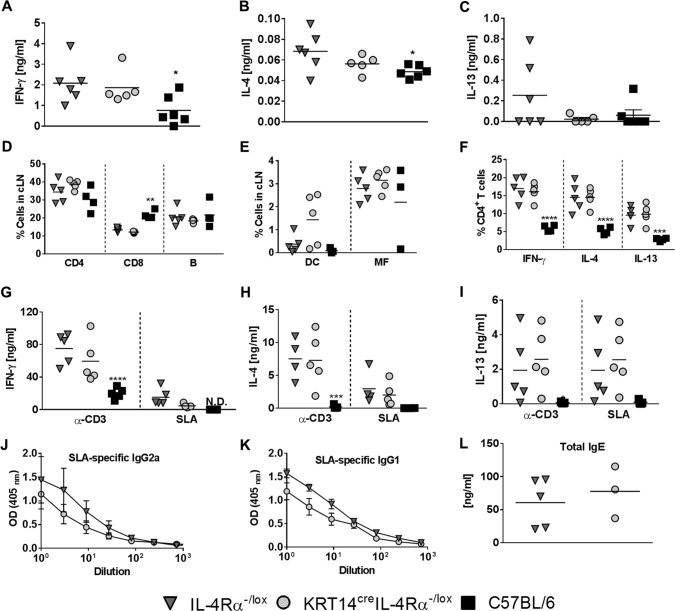
Similar immune responses between KRT14^cre^ IL-4Rα^−/lox^ BALB/c and littermate control IL-4Rα^−/lox^ mice infected with L. major LV39 in the ear dermis. (A to C) Early cytokine expression in the ears of mice infected with L. major LV39 at 2 weeks postinfection, with ELISA for IFN-γ (A), IL-4 (B), and IL-13 (C). At 8 weeks postinfection, mice were sacrificed and cervical draining lymph nodes (cLN) retrieved. Single-cell suspensions obtained from the lymph nodes were extracellularly stained for CD4 (CD3^+^CD4^+^) T cells, CD8 (CD3^+^CD8^+^) T cells, and B cells (CD19^+^B220^+^) (D), DC (dendritic cells) (CD11c^hi^ MHCII^hi^) and MF (macrophages) (CD11b^hi^ CD11c^−^ MHCII^hi^) (E), and stimulated with PMA-ionomycin-monensin, followed by staining for intracellular cytokine production (F). Cells were restimulated with anti-CD3 and SLA for 72 h, after which cytokine production was measured by ELISA for IFN-γ (G), IL-4 (H), and IL-13 (I). (J) L. major-specific type 1 (IgG2a) and (K and L) type 2 antibodies (IgG1 and total IgE) were measured by ELISA of sera from mice infected for 6 weeks. Data are representative of two experiments (*n* = 3 to 5 mice per group). Statistical analysis was performed with comparisons to the control IL-4Rα^−/lox^ littermate mouse group as significant (*, *P* < 0.05; **, *P* < 0.01; ***, *P* < 0.001; ****, *P* < 0.001), using 2-way ANOVA with Dunnett’s multiple-comparison test (A to I), two-way ANOVA with Bonferroni posttests (J and K), and Mann-Whitney test (nonparametric, unpaired *t* test) (L).

The early source of IL-4 can contribute to susceptibility to L. major infection (13). Thus, we measured IL-4 expression directly in the skin of L. major-infected mice. Here, mice were infected with L. major LV39 in the ear dermis, and at 2 weeks postinfection the ears were retrieved and processed for the detection of IFN-γ, IL-4, and IL-13 at mRNA and protein levels (Fig. 5A to C). IFN-γ, IL-4, and IL-13 were found to be undetectable at the mRNA level, as measured by quantitative PCR (qPCR) (data not shown). Detection of IFN-γ, IL-4, and IL-13 by ELISA showed no significant differences between control IL-4Rα^−/lox^ BALB/c littermates and KRT14^cre^ IL-4Rα^−/lox^ BALB/c mice in the L. major LV39-infected ears (Fig. 5A to C). However, IFN-γ and IL-4 levels in the ears were significantly decreased in C57BL/6 mice compared to those in littermate control IL-4Rα^−/lox^ BALB/c mice (Fig. 5A and B). Additionally, at week 1 postinfection, IL-4 was not detectable at the mRNA or protein level in the ears of KRT14^cre^ IL-4Rα^−/lox^ BALB/c mice infected with L. major LV39 (data not shown). While IL-4 was not detectable at the time analyzed, this finding suggests that it is produced locally or at very low doses that are not detectable. Additionally, IL-4 was reported to be produced at an earlier time and produced at low quantities at this earlier time point (13).

### Cellular and humoral immune responses of KRT14^cre^ IL-4Rα^−/lox^ BALB/c mice remain unaffected by the deletion of IL-4Rα on keratinocytes following L. major infection into the ear.

Following infection with L. major LV39 for 8 weeks, cell populations infiltrating the draining cervical lymph nodes were similar between KRT14^cre^ IL-4Rα^−/lox^ BALB/c and littermate control IL-4Rα^−/lox^ BALB/c mice (Fig. 5D and E). CD4^+^ T helper cytokine production by intracellular staining of cervical lymph node cells (Fig. 5F) and total cell cytokine production by lymph node restimulation (Fig. 5G to I) demonstrated similar levels of IFN-γ, IL-4, and IL-13 between KRT14^cre^ IL-4Rα^−/lox^ BALB/c and littermate control IL-4Rα^−/lox^ BALB/c mice. C57BL/6 mice exhibited heightened CD8^+^ T cell production in lymph nodes (Fig. 5D) and reduced IFN-γ, IL-4, and IL-13 production (Fig. 5F to I) compared to IL-4Rα^−/lox^ BALB/c mice. No differences in type 1 and type 2 antibody titers were observed in the sera of KRT14^cre^ IL-4Rα^−/lox^ BALB/c mice compared to littermate control IL-4Rα^−/lox^ BALB/c mice (Fig. 5J to L).

**FIG 6:**
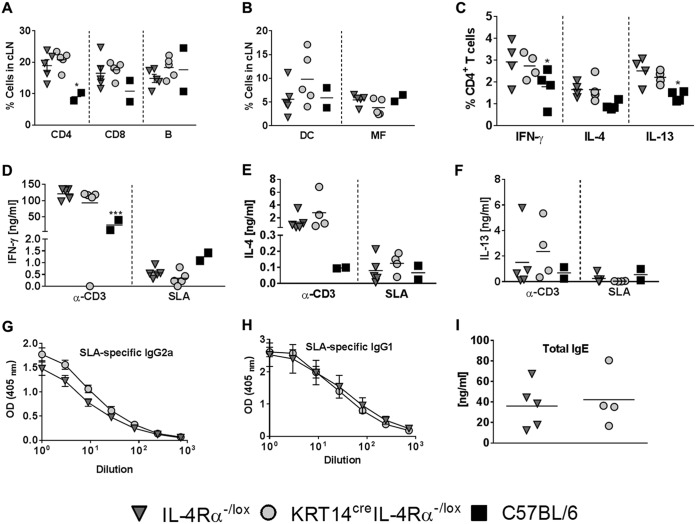
Low-dose infection in the ear dermis does not significantly alter immune response between control littermates and KRT14^cre^ IL-4Rα^−/lox^ mice on a BALB/c genetic background during L. major IL-81 infection. At 8 weeks postinfection, mice were sacrificed and cervical draining lymph nodes (cLN) retrieved. Single-cell suspensions obtained from the lymph nodes were extracellularly stained for CD4 (CD3^+^CD4^+^) T cells, CD8 (CD3^+^CD8^+^) T cells, and B cells (CD19^+^ B220^+^) (A), DC (dendritic cells) (CD11c^hi^ MHCII^hi^) and MF (macrophages) (CD11b^hi^ CD11c^−^ MHCII^hi^) (B), and also stimulated with PMA-ionomysin-monensin (C), followed by staining for intracellular cytokine production. Cells were restimulated with anti-CD3 and SLA for 72 h, after which cytokine production was measured with ELISA for IFN-γ (D), IL-4 (E), and IL-13 (F). (G to I) L. major-specific type 1 (IgG2a) (G) and type 2 antibodies (IgG1 and total IgE) (H and I) were measured by ELISA of sera from mice infected for 6 weeks. Data are representative of two experiments (*n* = 3 to 5 mice per group). Statistical analysis was performed with comparisons to the control IL-4Rα^−/lox^ littermate mouse group as significant (*, *P* < 0.05; ***, *P* < 0.001), using 2-way ANOVA with Dunnett’s multiple-comparison test (A to F), two-way ANOVA with Bonferroni posttests (G and H), and Mann-Whitney test (nonparametric, unpaired *t* test) (I).

L. major IL-81 infection of KRT14^cre^ IL-4Rα^−/lox^ BALB/c mice illustrated similar frequencies of cervical lymph node cell populations (Fig. 6A and B), cytokine production (Fig. 6C to F), and antibody titers (Fig. 6G to I) compared to levels for littermate control IL-4Rα^−/lox^ BALB/c mice. C57BL/6 mice infected with L. major IL-81 had significantly lower CD4^+^ T helper infiltration (Fig. 6A), significantly reduced CD4^+^ T cell-produced IFN-γ and IL-13 (Fig. 6C), and significantly reduced total cell IFN-γ (Fig. 6D) compared to levels for the littermate control IL-4Rα^−/lox^ BALB/c mice.

Together, these findings highlight that during intradermal infection in the ear, IL-4Rα signaling on keratinocytes in BALB/c mice does not affect protective immune responses, as determined by cellular responses, cytokine production, and type 1 and type 2 antibody titers.

## DISCUSSION

Previous studies have supported a role for IL-4 in driving a polarized Th1 response and conferring resistance to BALB/c mice during L. major infection (8, 25). Conversely, during experimental cutaneous leishmaniasis in C57BL/6 mice with a global deletion of IL-4Rα, it was seen that the deletion had no impact on resistance in these mice (16). Dendritic cells are the primary source of IL-12 and initiate antigen-specific immunity to *Leishmania* (31). Biedermann et al. incubated bone marrow-derived DCs with a Th1-priming adjuvant in the presence of recombinant IL-4 and found increased IL-12 production upon IL-4 stimulation. Ova-specific CD4^+^ T cells primed with activated dendritic cells also had significantly upregulated IFN-γ under these conditions, indicating that IL-4 could instruct dendritic cells to secrete IL-12, thereby inducing Th1 cell differentiation (8). Biedermann et al. also demonstrated this IL-4 instruction theory in mouse studies, showing that exogenous IL-4 administered during the period of dendritic cell activation was required to drive dendritic cells to elicit the Th1 response in usually susceptible BALB/c mice (8). The study by Biedermann et al. highlighted that a high dose of IL-4 administered exogenously was needed to observe a shift to protective Th1 immunity; however, the study did not provide insight on physiological doses of endogenous IL-4 during infection. Hurdayal et al. (9) addressed this using BALB/c mice with abrogated IL-4Rα expression on CD11c^+^ dendritic cells. During L. major infection these mice were hypersusceptible to infection, showing increased footpad swelling and parasite loads and increased Th2 immune responses. This indicated that IL-4Rα-responsive dendritic cells play an important role in the elicited immune response. Ehrchen et al. further explored the IL-4 instruction theory by focusing on the microenvironment of the infected tissue (13). Importantly, various cytokines, including IL-4, were induced in L. major-infected C57BL/6 mice and L. major-infected BALB/c mice (13). Additionally, cytokine induction, including IL-4 in the skin and by keratinocytes, was higher in C57BL/6 mice than in BALB/c mice, and genes involved in keratinocyte differentiation were found to be overrepresented, suggesting an influence on their expression by the presence of L. major parasites. While Biedermann et al. showed that exogenous recombinant IL-4 could drive dendritic cells to instruct a Th1 response (8), Ehrchen et al. illustrated that blocking endogenous IL-4 with exogenous anti-IL-4 antibody in L. major-infected C57BL/6 mice caused these mice to switch from a genetically resistant Th1 to a susceptible Th2 phenotype (8). Following on from Biedermann et al. (8) and Ehrchen et al. (13), we aimed to investigate whether IL-4Rα-responsive keratinocytes influence the outcome of disease during CL. Our initial study showed that in genetically resistant C57BL/6 mice, IL-4Rα-responsive keratinocytes did not affect the resistant phenotype (16), as seen by Ehrchen et al. in these resistant mice (13). The next step was to investigate whether deletion of IL-4Rα-responsive keratinocytes would contribute to nonhealing disease during experimental CL in BALB/c mice. If IL-4Rα is indeed demonstrated to be important for disease outcome, IL-4/IL-13 released by these keratinocytes could signal and influence keratinocytes in an autocrine manner or influence dendritic cells in a paracrine manner. A keratinocyte-specific IL-4Rα-deficient mouse model was generated using the Cre/*loxP* system under the control of the *krt14* locus. These mice were infected with L. major either s.c. in the footpad or i.d. in the ear, after which disease progression and cellular and humoral immunity were evaluated.

Subcutaneous infection of L. major IL-81 or LV39 in the footpad of KRT14^cre^ IL-4Rα^−/lox^ BALB/c mice revealed no role for IL-4Rα-responsive keratinocytes in modulating nonhealing disease to L. major. KRT14^cre^ IL-4Rα^−/lox^ BALB/c mice were susceptible to infection, showing progressive footpad swelling and parasite loads, characteristic of genetically susceptible BALB/c mice (32). L. major IL-81-infected KRT14^cre^ IL-4Rα^−/lox^ BALB/c mice showed dissemination of parasites to the spleen, similar to that of littermate control IL-4Rα^−/lox^ BALB/c mice and as expected in susceptible wild-type BALB/c mice (33, 34). In these mice, the mounting of a Th2 response, along with the alternative activation of macrophages, is the reason why parasites disseminate and cause severe disease (35–38). The parasites can use the polyamines generated from alternative activation of macrophages for their growth and survival (9). Disease progression in BALB/c mice infected with L. major corresponds to an upregulation of Th2 cytokines, CD4^+^ Th2 cells, and type 2 antibody responses (9, 26, 39–42). Increased infiltration of macrophages to the draining lymph node in C57BL/6 mice could be linked to the roles they play in protection, being essential not just for uptake of parasites and establishment of an immune response but also for their killing function by classically activated macrophages (1, 6, 43–45). Together, the data suggested that BALB/c mice with abrogated IL-4Rα were susceptible to L. major infection and that the absence of IL-4Rα expression on keratinocytes did not allow for protection against L. major.

An intradermal inoculation of low-dose stationary-phase promastigote L. major parasites in the ear better mimics natural infection by the sandfly than s.c. inoculation (28–30, 46–49). KRT14^cre^ IL-4Rα^−/lox^ BALB/c mice infected i.d. with 10^4^ L. major parasites showed swelling progression and parasite burden similar to that of littermate control IL-4Rα^−/lox^ BALB/c mice. L. major LV39-infected KRT14^cre^ IL-4Rα^−/lox^ BALB/c mice showed significantly fewer parasites at the site of infection (the ear) than the littermate control IL-4Rα^−/lox^ BALB/c mice. This suggests that KRT14^cre^ IL-4Rα^−/lox^ BALB/c mice were able to control parasite replication in the ear and that IL-4/IL-13 signaling through IL-4Rα on keratinocytes hampers immune regulatory functions when present. Upon investigation of the early production of IL-4 in the ear after 2 weeks of infection with L. major LV39, we found no significant change in KRT14^cre^ IL-4Rα^−/lox^ BALB/c mice compared to results for littermate control IL-4Rα^−/lox^ BALB/c mice, while resistant C57BL/6 mice appeared to have significantly lower production of IL-4. Overall, our data suggested that while IL-4Rα-responsive keratinocytes were not contributing to the development of the lesions or overall T helper immune responses of KRT14^cre^ IL-4Rα^−/lox^ BALB/c mice in the ear infection model, parasite replication at the site of the L. major LV39 infection was more controlled in these mice than in littermate control IL-4Rα^−/lox^ BALB/c mice.

Hence, our findings indicate that while IL-4/IL-13 signaling via IL-4Rα on keratinocytes in BALB/c mice does not contribute to their nonhealing phenotype, it could contribute to parasite control or replication at the site of infection in the ear model during L. major LV39 infection but not during L. major IL-81 infection. This finding needs to be further explored. This study therefore provides some understanding of the early immune responses occurring in the dermis and during cutaneous leishmaniasis in nonhealer BALB/c mice, and it also complements our recent findings in healer C57BL/6 mice (16). Additionally, our data highlight the importance of the strain of the L. major parasite, as well as the use of a more physiological route of infection, when studying cutaneous leishmaniasis in experimental mouse models.

## MATERIALS AND METHODS

### Ethical statement.

All mice were kept under specific-pathogen-free conditions. Mouse experiments were performed in strict accordance with the South African National Standard (SANS 10386:2008), as well as with the Animal Research Ethics Committee of the Faculty of Health Sciences, University of Cape Town (license no. 015/034).

### Generation and genotyping of KRT14^cre^ IL-4Rα^−/lox^ mice.

Keratinocyte cell-specific IL-4Rα-deficient (KRT14^cre^ IL-4Rα^−/lox^) BALB/c mice were generated using the Cre/*loxP* system and characterized by our laboratory. Briefly, KRT14^cre^ mice (Jackson Laboratory) were crossed with IL-4Rα^−/−^ BALB/c mice (17) and transgenic IL-4Rα^lox/lox^ mice (18) to generate hemizygous KRT14^cre^ IL-4Rα^−/lox^ BALB/c mice after nine generations of breeding. All mice were kept under specific-pathogen-free conditions in individually ventilated cages. Experimental mice were age and sex matched and used between 8 and 10 weeks of age. Genotyping of KRT14^cre^ IL-4Rα^−/lox^ BALB/c mice was carried out using the following specific primers: KRT14 P1, forward primer, 5′-TTC CTC AGG AGT GTC TTC GC; KRT14 P2, reverse primer, 5′-GTC CAT GTC CTT CCT GAA GC; KRT14 P3, forward primer, 5′-CAA ATG TTG CTT GTC TGG TG; KRT14 P4, reverse primer, 5′-GTC AGT CGA GTG CAC AGT TT.

### Functional characterization of KRT14^cre^ IL-4Rα^−/lox^ mice.

IL-4Rα deletion was confirmed with flow cytometry, with staining for IL-4Rα on ear-isolated keratinocytes. Briefly, ears were digested in complete Dulbecco’s modified Eagle’s medium (cDMEM) containing 0.2 mg/ml Liberase (TL research grade; Roche) and filtered through a 40-µm filter. Keratinocytes were then isolated, counted, and labeled. Labeling was performed by adding the antibodies for 20 min on ice, followed by the addition of the secondary antibody. Keratinocytes were gated as CD45^−^ and were double positive for CD49 and K14. Antibodies included CD45-peridinin chlorophyll protein (PerCP), CD49f-fluorescein isothiocyanate (FITC), mouse anti-keratin 14, and goat anti-mouse A555 (ThermoFisher Scientific). CD45^−^ CD49^+^ K14^+^ keratinocytes were then stained for the presence of IL-4Rα with IL-4Rα-phycoerythrin (PE) (BD, Pharmingen), and samples were acquired on a Fortessa machine (BD, San Jose, CA, USA). Flow data were analyzed using FlowJo software (TreeStar, Ashland, OR, USA). Cell surface IL-4Rα expression was further analyzed in total lymphocytes, Th cells (CD3^+^ CD4^+^), B cells (CD19^+^ B220^+^), dendritic cells (CD11c^+^ MHCII^+^), and macrophages (CD11b^+^ MHCII^+^) in the draining lymph node of L. major-infected IL-4Rα^−/−^, IL-4Rα^−/lox^, and KRT14^cre^ IL-4Rα^−/lox^ mice by flow cytometry. For *Dsc-1* mRNA expression in keratinocytes, primary keratinocytes were isolated from the tail skin of adult mice. Briefly, the skin from the tail was collected and incubated at 4°C for 16 h in 5 U/ml dispase (STEMCELL) supplemented with 1% penicillin-streptomycin-neomycin (PSN) (Gibco) and 0.5% gentamicin (Gibco). The epidermis was next separated from the dermis and treated with 0.2% trypsin (Gibco) for 5 min at 37°C. The reaction was stopped with fetal calf serum (FCS), and cells were then collected by crushing the epidermis through a 100-µm cell strainer. A total of 0.75 × 10^6^ cells/ml were plated in a 6-well plate coated with type 1 collagen (STEMCELL). Cells were grown for 8 days at 37°C in CnT-57.S medium (CELLnTEC) supplemented with 1% PSN and 0.5% gentamicin. The medium was discarded and cells were detached with 200 µl 0.2% trypsin for 5 min at 37°C. Cells were counted and plated at 0.3 × 10^6^ cells/ml in a 96-well plate coated with type 1 collagen. Isolated keratinocytes were then stimulated with 20 ng/ml of mouse IL-4 recombinant protein (Affymetrix, eBioscience) or left untreated for 24 h at 37°C as previously described (22). mRNA was isolated thereafter using an RNA Minikit (Qiagen) according to the manufacturer’s instructions, cDNA synthesized, and quantitative reverse transcription-PCR (RT-PCR) performed using a LightCycler (Roche). Values were normalized to *hprt* level and are presented as fold induction compared to the level for unstimulated keratinocytes. The primer sequences for *Dsc-1* were the following: forward (F), 5′-GGGAGCACCTTCTCTAAGCA-3′; reverse (R), 5′-TTTTGACAGGCATCACAAAATAA-3′ (22).

### L. major infection.

L. major LV39 substrain 50132 (MRHO/SU/59/P), obtained from the American Type Culture Collection (ATCC), an LV39 substrain (MRHO/SV/59/P) obtained from the University of Lausanne (50, 51), and L. major IL-81 (MHOM/IL/81/FEBNI) were maintained via continuous passage in BALB/c mice (17), and *in vivo* cultures were incubated in Schneider’s medium (Sigma-Aldrich) supplemented with 20% FCS in a T25 tissue flask (Corning). Parasites were prepared for infection as previously described (17). Mice were anesthetized prior to subcutaneous inoculation with 2 × 10^5^ (IL-81) or 2 × 10^6^ (LV39) stationary-phase promastigotes into the left hind footpad, contained in a volume of 50 µl of phosphate-buffered saline (PBS) (9, 17, 39). Disease progression was monitored weekly by measuring change in swelling of infected footpads using a Mitutoyo micrometer caliper (Brütsch, Zürich, Switzerland). Alternatively, mice received an intradermal inoculation with 1 × 10^4^ stationary-phase promastigotes in the left ear, contained in a volume of 10 µl of PBS (28–30). Disease progression was monitored weekly by measuring change in diameter of lesions of the infected ear using a digital Vernier caliper (South Africa).

### Detection of viable parasite burden.

Infected footpad, ear, draining lymph node, and spleen cell suspensions were cultured in Schneider’s culture medium (Sigma). Parasite burden was determined with the limiting dilution assay (LDA) that has been previously described (17).

### Early detection of IFN-γ, IL-4, and IL-13 in infected ears.

Mice were infected in the left ear with intradermal inoculation of 1 × 10^4^ stationary-phase promastigotes, contained in a volume of 10 µl of PBS as described above. At 2 weeks postinfection, infected and noninfected ears were retrieved for early detection of IFN-γ, IL-4, and IL-13 with qPCR. Whole ear was extracted and homogenized in QIAzol lysis reagent (Giagen, Germany). Total RNA was isolated from the homogenate using an RNeasy Minikit (Qiagen, Germany) according to the manufacturer’s instructions. RNA quantity and purity were measured with an ND-1000 NanoDrop (ThermoScientific, DE, USA). Reverse transcription was performed using a transcript first-strand cDNA synthesis kit (Roche, Germany) according to the manufacturer’s instructions. Real-time qPCR was performed using LightCycler 480 SYBR green I master mix (Roche, Germany) and gene-specific primers (IDT, CA, USA). The mRNA expression of each gene was normalized to the housekeeping gene encoding hypoxanthine phosphoribosyl transferase (HPRT). The primer sequences were the following: HPRT F, 5′-GTT GGA TAT GCC CTT GAC-3′; R, 5′-AGG ACT AGA ACA CCT GCT-3′; IL-4 F, 5′-TCG GCA TTT TGA ACG AGG TC-3′; R, 5′-GAA AAG CCC GAA AGA GTG GCA-3′; IL-13 F, 5′-CTC ACT GGC TCT GGG CTT CA-3′; R, 5′-CTC ATT AGA AGG GGC CGT GG-3′; IFN-γ F, 5′-GCT CTG AGA CAA TGA ACG CT-3′; R, 5′-AAA GAG ATA ATC TGG CTC TGC-3′. For ELISA detection of the cytokines IFN-γ, IL-4, and IL-13, whole ear was extracted and homogenized in Tween saline buffer and ELISA was performed as described below.

### *Ex vivo* restimulation of draining lymph node cells.

Single-cell lymph node suspensions (1 × 10^6^) were stimulated with 20 μg/ml anti-CD3 or 50 μg/ml SLA and incubated at 37°C and 5% CO_2_ for 72 h. Supernatants were collected and cytokines were measured by sandwich ELISA as previously described (17).

### Flow cytometry.

Single-cell lymph node suspensions (1 × 10^6^ cells/well) were seeded (96-well Nunc plate) and stained for the expression of surface markers for lymph node cell populations (T cells, B cells, dendritic cells, and macrophages). The T and B cell panel included CD3-FITC, CD4-PE, CD8-allophycocyanin (APC), and CD19**-**PerCP-Cy5.5. The dendritic cell and macrophage panel included CD11c-APC, CD11b-PE, and major histocompatibility complex class II (MHCII)-FITC. Each mix also included 1% rat serum and 10 µg Fc gamma receptor blocker (*Fc*γ). For intracellular cytokine staining, single-cell lymph node suspensions (2 × 10^6^ cells/well) were stimulated at 37°C for 2 h with 50 ng/ml PMA and 250 ng/ml ionomycin, followed by the addition of 200 μM monensin for 4 h. Cells were stained with extracellular markers (CD3-FITC, CD4-PerCP, CD8-APC, 1% rat serum, and 10 µg *Fc*γ), fixed with 2% paraformaldehyde, and permeabilized with 0.5% saponin buffer, followed by intracellular staining for IFN-γ, IL-4, and IL-13 with PE-labeled anti-mouse antibodies. Acquisition of cells was completed on a FACSCalibur machine (BD Immunocytometry Systems, San Jose, CA, USA). Flow data were analyzed using FlowJo software (Treestar, Ashland, OR, USA).

### ELISA for cytokine and antibody detection.

Cytokines were detected in the cell supernatants with a sandwich ELISA as previously described (17). Detection of serum antigen-specific levels of IgG2a and IgG1 and total IgE was performed as previously described (17). Microtiter plate readings were carried out with a VersaMax ELISA plate reader.

### Statistics.

Statistical analysis was carried out using GraphPad Prism 7 software. The data were calculated as means ± standard errors of the means (SEM). There was a normal distribution of samples, and statistical significance was determined with specific tests as stated for each experiment, defining differences from IL-4Rα^−/lox^ as significant (*, *P* < 0.05; **, *P* < 0.01; ***, *P* < 0.001; ****, *P* < 0.0001).
